# Mannose-binding lectin gene variants and infections in patients receiving autologous stem cell transplantation

**DOI:** 10.1186/1471-2172-15-17

**Published:** 2014-05-03

**Authors:** Ana Moreto, Concepción Fariñas-Alvarez, Maria Puente, Javier Gonzalo Ocejo-Vinyals, Pablo Sánchez-Velasco, Juan Pablo Horcajada, Ana Batlle, Carmen Montes, Francisca Santos, Eulogio Conde, Maria-Carmen Fariñas

**Affiliations:** 1 Department of Hematology, Hospital Universitario Marqués de Valdecilla, University of Cantabria, IFIMAV, Santander, Spain; 2 Health Care Quality Unit, Hospital Universitario Marqués de Valdecilla, University of Cantabria, IFIMAV, Santander, Spain; 3 Department of Immunology, Hospital Universitario Marqués de Valdecilla, University of Cantabria, IFIMAV, Santander, Spain; 4 Infectious Diseases Unit, Hospital Universitario Marqués de Valdecilla, University of Cantabria, IFIMAV, Santander, Spain; 5 Current address: Department of Hematology, Hospital Universitario de Cruces, Bilbao, Spain; 6 Current address: Department of Infectious Diseases, Hospital Universitari del Mar, Barcelona, Spain

**Keywords:** MBL, Gene variant, Polymorphism, Infection, Autologous stem cell transplant

## Abstract

**Background:**

Serious infections are common in patients undergoing autologous stem cell transplantation (ASCT) mainly because of the effects of immunosuppression. The innate immune system plays an important role in the defense against different infections. Mannose binding lectin (MBL) is a central molecule of the innate immune system. There are several promoter polymorphisms and structural variants of the *MBL2* gene that encodes for this protein. These variants produce low levels of MBL and have been associated with an increased risk for infections.

**Methods:**

Prospective cohort study. The incidence, severity of infections and mortality in 72 consecutive patients with hematologic diseases who underwent ASCT between February 2006 and June 2008 in a tertiary referral center were analyzed according to their *MBL2* genotype. INNO-LiPA *MBL2* was used for *MBL2* gene amplification and genotyping. Relative risks (RR) (IC95%) as measure of association were calculated. Multivariate analysis was performed using logistic regression.

**Results:**

A statistically significant higher number of fungal infections was found in patients with *MBL2* variants causing low MBL levels (21.1%versus1.9%, p=0.016). In this *MBL2* variant group infection was more frequently the cause of mortality than in the *MBL2* wild-type group (p=0.05). Although not statistically significant, there was a higher incidence of major infections in the *MBL2* variant group as well as a higher number of infections caused by gram-positive bacteria.

**Conclusions:**

Low-producer *MBL2* genotypes were associated with an increased number of fungal infections in ASCT patients, which would suggest that MBL has a protective role against such infections. ASCT patients with *MBL2* variant genotypes are more likely to die as a result of an infection.

## Background

Patients undergoing autologous stem cell transplantation (ASCT) are at a high risk of serious infection, mainly due to both the procedure and the underlying disease requiring the transplant, which in most patients undergoing the procedure is hematological [1-5].

Mannose-binding lectin (MBL) is a circulating liver-synthesized C-type serum protein of importance in the innate immune defense system. It is one of the recognition molecules in the lectin complement activation pathway [6]. Human MBL is derived from a single gene located on chromosome 10 (*MBL2*) [3-8].

Serum levels and the function of MBL oligomers are strongly influenced by genetic variants [9]. Low levels of MBL have been associated with increased susceptibility to infections, especially in recurrent or severe forms, even in immunocompetent individuals [10]. However, isolated low MBL serum levels do not usually cause disease. This deficiency appears to be associated with severe infections when other immune deficiencies occur [11].

The effect of MBL deficiency in the infectious complications of patients receiving chemotherapy is variable [11]. It has been associated with serious infections and more prolonged episodes of febrile neutropenia [11-13], as well as with an earlier first severe infection in deficient patients [14]. There are also several studies which analyze the relationship between MBL and allogeneic stem cell transplantation infections [15-22], but until now, only two studies dealing with this issue in ASCT have been published [23,24]. The focus of this study is the association between *MBL2* gene variants and the risk of infections in patients with different hematologic diseases undergoing ASCT.

### Patients and methods

#### Patients

A prospective cohort study of all patients who received an ASCT for hematologic disease from February 2006 to June 2008 in the Hospital Universitario Marqués de Valdecilla (1,000-bed teaching hospital) was performed. All transplanted patients >13 years old without immunodeficiency (solid tumors, HIV infection or congenital immunodeficiencies) were included. Patients were followed up after inclusion in the study for 6 months.

#### Infections

Intercurrent infections were monitored during the early period (0–30 days after ASCT), the intermediate period (30–100 days after ASCT) and the late period (>100 days after ASCT). Infections were prospectively collected by the clinical research associates from the institution using a standardized data collection form, following the protocols of the Hematology Department of the Hospital Universitario Marqués de Valdecilla, in compliance with the clinical guidelines of the Infectious Diseases Society of America [25]. Prophylactic measures included isolation in high-pressure rooms with air filters and the administration of intravenous acyclovir at prophylactic doses from day -3. Antibiotics and antifungal prophylaxis were not routinely administered.

An infectious episode was defined as a microbiologically or clinically documented infection. Microbiologically documented infections with the same organisms during the same phase of therapy occurring more than 7 days apart were counted as different infections. Major infection was predefined as either sepsis confirmed on microbiological testing or a systemic inflammatory response syndrome with highly suggestive radiographic or clinical characteristics and the requirement for a specific antimicrobial treatment in both cases. The following conditions were excluded: single positive blood culture resulting from contaminant skin bacteria, upper respiratory tract infections, culture-negative interstitial pneumonitis, dermatological varicella zoster reactivation, and local herpes simplex reactivation. Severe herpes virus infection was predefined as invasive viral infections requiring prompt treatment and hospitalization. A positive cytomegalovirus (CMV) blood culture or CMV-pp65 antigenemia assay or polymerase chain reaction (PCR) for CMV DNA (>400 copies/ml) (COBAS Amplicor CMV Monitor, Roche Diagnostics) and symptoms of organ dysfunction were required to fulfill the criteria of a clinically significant CMV infection/reactivation [26]. Episodes of asymptomatic CMV reactivation, primary varicella zoster virus and Epstein-Barr virus infections were excluded.

#### MBL2 genotyping

Blood was collected in EDTA-stabilized tubes in compliance with approved protocols from our institution. Genomic DNA from patients and controls was extracted from peripheral blood by using the Maxwell 16 Genomic DNA Purification system. For *MBL2* gene amplification and genotyping, the INNO-LiPA *MBL2* (Innogenetics Diagnostica Iberia S.L.U, Barcelona, Spain) was used, following the manufacturer’s instructions. The INNO-LiPA *MBL2* is a line probe assay, designed for genotyping the 6 variations in the human *MBL2* gene (-550G > C, -221G > C, +4C > T, R52C, G54D, and G57E), providing an analysis of the seven common haplotypes and the 28 possible resulting diplotypes.

Exon mutations called structural variants (normal allele or “wild-type” *A* and mutant alleles *B*, *C* and *D* which together were called allele *O*) and promoter polymorphisms (*H/L*, *Y/X* and *P/Q*) were analyzed. Variants *YA/YA*, *XA/XA* and *YA/XA* were classified as *A/A*. Those with any mutation in the exon such as *YA/O*, *XA/O* were classified as *A/O* and, lastly, individuals homozygous for the structural variants in the exon were considered as *O/O*. Thus, patients were divided in two groups according to their *MBL2* genotype: 1) normal homozygous patients (*AA*) and 2) patients with *AO* and *OO* genotypes, who were considered to be lower MBL producers [24].

#### Statistical analysis

Results were analyzed according to *MBL2* genotype, comparing normal homozygous patients (*AA*) and patients with *AO* and *OO* genotype and to determine if there were differences between the two groups in terms of demographic characteristics, underlying disease, infection parameters and mortality. Statistical analysis was performed using a two-tailed *χ*^2^ test and a Fisher’s exact test, Student *t*-test or Mann Whitney, as appropriate in each case. For m × n tables, a Fisher’s exact test was estimated using the Monte Carlo method. Relative risks (RR) and their 95% confidence intervals (CI) were calculated to analyze the association between genetic variants and polymorphisms and the risk of concomitant infections as dependent variables (overall and by microorganisms). Multivariate analysis was performed using logistic regression to estimate the association between genetic variants and polymorphisms and the risk of underlying infections adjusted by confusion factors. A regression model was constructed for each type of infection, with infection-related variables being the outcome and the independent variable being the *MBL2* genotypes, adjusted for sex, age, hematologic disease, active pre-transplant hematologic disease. A two-tailed P <0.05 was considered statistically significant. Data were analyzed using Stata (SE 10.0, Stata Corporation, College Station, TX) and SPSS (version 19) (SPSS Inc., Chicago, IL) statistical software.

#### Ethics

This study was approved by the Ethics Committee of the Autonomous Community of Cantabria, Spain. All patients (>18 years) signed informed consent to participate in the study. For children under 18 years, the parents had to sign informed consent.

## Results

### ***MBL2*** structural variants, promoter polymorphisms and genotypes

Seventy-two patients were included. Median age at the time of transplantation was 54 years (19–74). During the study period, no patients under 19 years underwent AST. Forty-two (42) patients were men. Patients were divided in two groups according to their *MBL2* genotype: patients homozygous (*AA*) for wild type *MBL2* (n = 53) and other patients, *AO/OO* (n = 19), which included heterozygous patients (*AO*) (n = 16), and homozygous patients (*OO*) (n = 3). No statistically significant differences were found in both groups for gender, age, distribution according to different types of hematologic disease or hematologic disease activity at the time of transplantation. Patient characteristics, underlying disease and number of previous lines of treatment are shown in Tables 1 and 2.

**Table 1:** Characteristics and MBL genotypes of 72 patients with ASCT

	***AA Patients (%) n = 53***	***AO/OO Patients (%) n = 19***	P-value*
**Gender**			
**Male**	30 (56.6)	12 (63.2)	0.62
**Female**	23 (43.4)	7 (36.8)	
**Mean age (SD), years**	51.6 (13.9)	50.2 (14.8)	0.70
**Underlying disease**			0.78
MM	14 (26.4)	6 (31.6)	
HD	8 (15.1)	5 (26.3)	
NHL	22 (41.5)	7 (36.8)	
AMN	7 (13.2)	1 (5.3)	
PL	1 (1.9)	0	
Amyloidosis	1 (1.9)	0	
Active disease**			0.22
YES	11 (20.8)	7 (36.8)	
NO	42 (79.2)	12 (63.2)	

*Two-tailed Chi-squared test/Fisher exact test for categorical variables or two-tailed t-test for continuous variables.

**Active pre-transplant hematologic disease.

MM: multiple myeloma, HD: Hodgkin disease, NHL: non Hodgkin lymphoma, AMN: acute myeloid neoplasia, PL: prolymphocytic leukemia, ME: multiple sclerosis.

*AA* genotype: patients homozygous for wild-type structural allele (*A*).

*AO/OO* genotype: patients heterozygous or homozygous for the *B*, *C* or *D* alleles.

**Table 2:** Number of chemotherapy lines pre-transplant

	***AA Patients (%) n = 53***	***AO/OO Patients (%) n = 19***	P-value*
**0**	1 (1.9)	0	0.18
**1**	14 (26.4)	5 (26.3)	
**2**	26 (49.1)	10 (52.6)	
**3**	12 (22.6)	2 (10.5)	
**4**	0	2 (10.5)	
**Mean (SD)**	1.9 (0.8)	2.1 (0.9)	0.55

*Two-tailed Fisher’s exact test for categorical variables or two-tailed t-test for continuous variables.

*AA* genotype: patients homozygous for wild-type structural allele (*A*).

*AO/OO* genotype: patients heterozygous or homozygous for the *B*, *C* or *D* alleles.

### Infections

Episodes of infection were recorded from the first day after infusion (day +1) until the sixth month post-transplantation. The average number of episodes of infection per patient was 2 (SD = 1.3) with a minimum of 0 and a maximum of 8 (1 patient). A total of 138 episodes of infection were collected in 69 patients. Three (3) patients had no infections during follow-up (4.2%). Thirty-one (31) patients (43.1.%) had a single episode, 20 (27.8%) had 2 episodes, 11 (15.3%) 3 episodes, 4 (5.6%) 4 episodes, 2 (2.8%) 5 episodes and one patient (1.4%) had 8 episodes of infection.

There were 112 infections in the early period (from day 1 to 30), 17 in the intermediate period (days 31 to 100) and 9 in the late period (day 101 to the end of the study). An average of 1.74 (SD = 1.04) episodes of infection occurred in the wild-type *MBL2* group, and 2.42 (SD = 1.84) in the variant *MBL2* group, with a greater number of infections in the deficient group (at the limit of statistical significance, p = 0.051).

Table 3 shows infection characteristics. Sepsis was common (43.1%) and equally distributed between both groups, as was urinary tract infection which occurred at a lower frequency (11.1%) than expected. There was also a low incidence of pneumonia (5.6% of all patients), which occurred more frequently in the *MBL2* variant group, 10.5% versus 3.8%, RR: 2.8 (0.4-18.4), *p* = 0.28. Venous catheter infection occurred at a similar rate in both groups; 11.3% versus 10.5%, risk ratio (RR): 0.9 [95% confidence interval (CI) 0.2-4.2, *p* = 1.00].

**Table 3:** ***MBL2 *** **genotypes and infections in 72 patients with ASCT during the 6 months of follow-up**

	***AA***	***AO/OO***	**RR (95% CI)**	P-value*
	***Patients (%)***	***Patients (%)***		
	***n = 53***	***n = 19***		
***Types of Infection***				
-Pneumonia	2 (3.8)	2 (10.5)	2.8(0.4-18.4)	0.28
-Sepsis	22 (41.5)	9 (47.4)	1.1 (0.6-2.0)	0.66
-VCI	6 (11.3)	2 (10.5)	0.9 (0.2-4.2)	1.00
-UTI	5 (9.4)	3 (15.8)	1.7 (0.4-6.3)	0.43
***Major infection***	6 (11.3)	5 (26.3)	2.32 (0.80-6.74)	0.15
***Gram-positive infections***	14 (26.4)	9 (47.4)	1.8 (0.9-3.4)	*0.09*
*-S. epidermidis*	9 (17)	7 (36.8)	2.2 (0.9-5.0)	0.11
*-S. aureus*	1 (1.9)	0 (0)	--	1.00
*-Other*				
***Gram-negative infections***	19 (35.8)	6 (31.6)	0.9 (0.4-1.9)	0.74
***Viral infections***	1 (1.9)	1 (5.3)	2.8 (0.2-42.4)	0.46
-CMV	0 (0)	1 (5.3)	--	0.26
***Fungal infections***	1 (1.9)	4 (21.1)	11.5 (1.3-93.7)	*0.016*
***-*** *Candida*	1(1.9)	2 (10.5)	5.6 (0.5-58.1)	0.17
***-*** *Aspergillus*	0 (0)	1 (5.3)	--	0.26
*-Mucor*	0 (0)	1 (5.3)	--	0.26
***Nº deaths***	4 (7.5)	3 (15.8)	2.1 (0.5-8.5)	0.37
***Cause of death***				
-Infection	0	3	--	0.05
-Progression	3	0	--	
-Unknown (not infection)	1	0	--	

*Two-tailed Chi-squared test or Fisher exact test as corresponding.

RR: risk ratio; CI: confidence interval.

VCI: Venous catheter infection; UTI: Urinary tract infection.

*AA* genotype: patients homozygous for wild-type structural allele (*A*).

*AO/OO* genotype: patients heterozygous or homozygous for the *B*, *C* or *D* alleles.

Fifteen major episodes of infection were recorded: sepsis in 8 patients; pneumonia in 5 patients, including one pulmonary aspergillosis and one Mucor pneumonia; and 2 patients with severe diarrheal episodes (one caused by *Clostridium difficile*). A higher incidence of major episodes of infection was observed in the variant *MBL2* group, 26.3% versus 11.3% in the *MBL2* wild-type group: RR = 2.32, (95% CI: 0.80-6.74, *p* = 0.15), although this did not reach statistical significance.

There were 68 microbiologically documented infections (50% of all the infections) in 39 patients. Fifty-six (82.4%) of these infections occurred in the early period, 5 (7.4%) in the intermediate and 7 (10.2%) in the late period. There were 33 infections caused by gram-positive bacteria, 36 caused by gram-negative bacteria, 5 fungal infections and 2 infections caused by virus. Eight were polymicrobial infections.

The average number of microbiologically documented infections was not statistically significant different between both groups, although there was a greater number of infections with microbiological documentation in the variant *MBL2* group: 1.32 (SD = 1.53) versus 0.81 (SD = 0.96 *p =* 0.1).

There was a higher average of microbiologically documented episodes of infection in the early period in the variant *MBL2* group (1.90 (SD = 1.2), than in the *MBL2* wild-type (1.37 (SD = 1.2 *p =* 0.062), but this did not reach statistical significance.

The average time in days until the first infection after the transplantation day was 7.98 days (SD = 11.34) in the wild-type *MBL2* group and 13 days (SD = 30.73) in the *MBL2* variant group (*p* = 0.32).

Regarding the type of infection, gram-positive bacteria were more frequent in the *MBL2* variant group, 47.4% versus 26.4%, RR = 1.8 (95% **CI**: 0.9-3.4, *p =* 0.09). There was a greater incidence of infections caused by *Staphylococcus epidermidis* in the *MBL2* variant group, 36.8% versus 17%, RR = 2.2 (95% **CI**: 0.9-5.0, *p =* 0.11). In the adjusted analysis, the odds ratio (OR) was 4.0 (95% **CI**: 1.0-16.5, *p* = 0.05), at the limit of statistical significance (Table 4). There was only 1 case of infection by *Staphylococcus aureus*, in the *MBL2* wild-type group.

**Table 4:** **Association between *MBL2 *** **genotypes and infections: Multivariate analysis**

**Outcome variable (patients with infection)**	OR* (95% CI) (Effect of *MBL2 * **genotypes on risk of infection)**	***p-value***
***Types of infection***		
-Pneumonia	8.1 (0.4-158.2)	0.17
-Sepsis	0.9 (0.3-3.1)	0.88
-VCI	0.8 (0.1-5.0)	0.77
-UTI	2.3 (0.4-12.8)	0.44
***Serious infection***	0.8 (0.1-4.9)	0.79
***Gram-positive infections***	2.5 (0.8-7.5)	0.11
*-S. epidermidis*	4.0 (1.0-16.5)	0.05
***Gram-negative infections***	0.7 (0.2-2.4)	0.59
***Viral infections***	1.1 (0.01-60.4)	0.96
***Fungal infections***	12.9 (1.1-153.9)	0.03

*Odds Ratio (OR) adjusted by: sex, age, type of hematologic disease, active pre-transplant hematologic disease.

CI: confidence interval.

VCI: Venous catheter infection; UTI: Urinary tract infection.

Most infections caused by gram-positive bacteria occurred in the early period and were more frequent in the variant *MBL2* group (42.1% versus 26.4% in the *MBL2* wild-type group, *p* = 0.2). There were no such infections in the intermediate period and only one in each group in the late period.

The incidence of fungal infections in the variant *MBL2* group (3 *Candida albicans*, 1 *Mucor*, 1 *Aspergillus*) was statistically significantly higher than in the *MBL2* wild-type group (1 *Candida* spp), RR = 11.5 (95% CI: 1.3-93.7, p = 0.016), as confirmed in a multivariate analysis (Table 4).

There were no statistically significant differences in the incidence of infection caused by gram-negative bacteria, RR: 0.9 (95% **CI**: 0.4-1.9, *p =* 0.74), or by virus between both groups, RR: 2.8 (95% **CI**: 0.2-42.4, *p* = 0.46), although there was a higher incidence of viral infections in the variant *MBL2* group.

More patients died in the variant *MBL2* group than in the wild-type *MBL2* group (15.8% versus 7.5% respectively, *p = 0.37*), although this difference was not statistically significant. However, all deaths in the variant *MBL2* group were related to infection, whereas in the wild-type *MBL2* group, deaths were due to other causes, with a *p*-value of 0.05 at the limit of statistical significance (Table 3).

## Discussion

This prospective study focused on comparing the incidence of infection and mortality after ASCT between patients with the wild-type *MBL2* genotype compared to patients carrying a variant *MBL2* genotype. The *MBL2* variant is associated with lower levels of serum MBL, so it may be related with a greater number of infections and/or more serious infections. A multivariate analysis was performed to rule out other factors that could cause a higher incidence or severity of infection (disease status-response *vs*. type of hematologic disease, active pre-transplant hematologic disease, sex and age).

The study group was homogeneous, with no differences in age, gender or hematologic disease, and presented similar disease activity at the time of transplantation.

So far, there are few studies describing the incidence of infections in ASCT related to *MBL2* gene variants. Horiuchi et al*.*[23] in a series of 113 patients treated with high-dose chemotherapy and ASCT, most of whom had hematologic diseases, carried out a study of the incidence of major bacterial infection in patients with low-producing genotypes compared to the wild-type genotype, finding an association between the low-producing genotypes and a highly increased and significant risk of major bacterial infections. The incidence of major bacterial infections (9%) was lower than that observed in the current study (18%). This could be related with the use of prophylactic antimicrobials including ciprofloxacin and fluconazole in all their patients, in contrast to the usual practice in our center.

Mølle *et al*. [24], in a retrospective study compared *MBL2* gene variants with the risk of severe infections in multiple myeloma in patients receiving ASCT after high-dose melphalan. They found that patients homozygous for wild-type *MBL2* had a significantly reduced risk of septicemia compared to patients carrying the variant *MBL2*. In our series, no differences were found in the number of patients with sepsis between the two groups. Although the lack of association between these infections and *MBL2* gene variants could be explained by the sample size, this could also be due to the different underlying diseases: in Mølle’s study, all patients were diagnosed with multiple myeloma while our patients had heterogeneous hematologic diseases and therefore could have had a higher risk of infection. They also found more severe infections in patients with variant *MBL2*, although the differences were not statistically significant. Similarly, in our series more severe infections and a greater number of episodes of pneumonia were found in patients with variant *MBL2*.

When the incidence of infections caused by different microorganisms was analyzed, a higher number of infections caused by gram-positive bacteria was found in patients carrying the variant *MBL2* form, and these were mainly staphylococci. In this respect, the incidence of infections caused by *Staphylococcus epidermidis* was greater in the *MBL2* variant group. These findings contrast with the similar number of central venous catheter infections found in both groups. This could mean that the increased number of infections caused by *S. epidermidis* is not only due to catheter infection. ASCT patients carrying the variant *MBL2* genotype, with MBL deficiency, may develop more *S. epidermidis* infections. However, the mechanism for this difference is unclear. A study by Hellemann et al. [27] in critically ill patients admitted to an intensive care, showed an association between the *MBL2 O/O* genotype with an increased incidence of gram-positive infections. However, in two different studies by Neth et al. [28] and Shang et al. [29], *S. epidermidis* generally demonstrated low MBL binding activity, which would contradict the hypothesis that MBL plays an important role in infections caused by *S. epidermidis*. One explanation that has been proposed for the diversity between the different studies may rely on the fact that MBL also functions as a scavenger molecule in maintaining internal tissue homeostasis. Apparent MBL associations may be due to disturbances in this scavenger system, rather than a direct antiinfectious effect [30].

In the current study, no differences were found in the incidence of infections caused by gram-negative bacteria in patients with wild-type *MBL2* genotype *versus* patients carrying a variant *MBL2* genotype. This is consistent with the studies by Hellemann [27] and Sutherland [31]. There was only one case of viral infection in each group; in the *MBL2* variant group this was caused by CMV, a potentially very serious disease, while the virus in the *MBL2* wild-type group was a reactivation of varicella zoster, which is more common in hematological patients.

Granell et al. [18] showed that MBL pathway dysfunction due to genetically determined deficiencies of MBL influence the outcome of allogenic stem cell transplantation by increasing susceptibility to fungal invasive infections. In patients with ASCT, Horiuchi et al. [23] found that 3 of the 16 patients with major infections who had a pulmonary aspergillosis were homozygous for *A*, i.e. the normal *MBL* allele. On the contrary, in our study, 4 patients with fungal infection carried the *MBL2* deficient genotype; 2 had *Aspergillus* and *Mucor* pneumonia (both died as a consequence of their fungal infection); 1 had candidemia; and 1 had disseminated mucocutaneous *Candida* infection. Several studies have been published showing that MBL may play a crucial role in the innate immunity against yeast infections by increasing PMN uptake [32,33].

In our series, the incidence of mortality due to infection was higher in the *MBL2* variant form, and the cause of death in all patients was major infection. These findings are in line with other groups of immunosuppressed patients who had low-producing *MBL2* genotypes [27,34].

## Conclusions

In summary, the results of our study suggest that ASCT patients with the *MBL2* variant gene have increased fungal infections, which would suggest that MBL offers protection against such infections. It would be reasonable to assess whether early antifungal prophylaxis in our ASCT patients with the *MBL2* variant gene could reduce the incidence of such infections. Due to the contradictory results of the previous reports, further studies are needed to clarify whether low-producing *MBL2* genotypes could play a role in gram-positive infections, especially in *S. epidermidis* infections. ASCT patients with *MBL2* variant genotypes are more likely to die as a result of an infection.

## Abbreviations

ASCT: Autologous stem cell transplantation; MBL: Mannose binding lectin; CMV: Cytomegalovirus; RR: Relative risk; CI: Confidence interval; OR: Odds ratio.

## Competing interest

The authors declare that they have no competing interests.

## Authors’ contributions

AM participated in data collection and manuscript preparation. CF-A participated in the design of the study, performed the statistical analysis and reviewed and revised the manuscript. MP participated in collection of data and manuscript preparation. JGO-V carried out part of the *MBL2* genotyping, and revised the manuscript. PS-V carried out part of the *MBL2* genotyping and revised the manuscript. JPH participated in the design of the study and reviewed and revised the manuscript. AB participated in collection of data. CM participated in collection of data and manuscript preparation. FS participated in collection of data and manuscript preparation. EC reviewed and revised the manuscript. MCF: participated in the design of the study, data analysis and reviewed and revised the manuscript. All authors read and approved the final manuscript.

## Authors’ information

Drs. Fariñas-Alvarez, Ocejo-Vinyals, Horcajada, Conde and Fariñas share senior authorship in this study.
